# Pre-TAVI imaging: an Italian survey by the CT PRotocol optimization (CT-PRO) group

**DOI:** 10.1007/s10554-024-03052-8

**Published:** 2024-01-24

**Authors:** Tommaso D’Angelo, Ludovica R. M. Lanzafame, Carlo Liguori, Cesare Mantini, Vincenzo Russo, Pierpaolo Palumbo, Giovanni B. Scalera, Andrea Iozzelli, Andrea Borghesi, Gildo Matta, Fabio Greco, Valeria Garretto, Massimiliano Danti, Roberto Iezzi, Marco Francone

**Affiliations:** 1 Unit of Diagnostic and Interventional Imaging, Department BIOMORF, University of Messina, Via Consolare Valeria 1, Messina, 98100 Italy; 2 Department of Radiology and Nuclear Medicine, Erasmus MC, Rotterdam, The Netherlands; 3 Radiology Unit, Ospedale del Mare, ASL NA1 Centro, Napoli, Italy; 4 Department of Neuroscience, Imaging and Clinical Sciences, “Gabriele D’Annunzio” University of Chieti-Pescara, Chieti, Italy; 5 Pediatric and Adult CardioThoracic and Vascular, Oncohematologic and Emergency Radiology Unit, Radiology Department IRCCS, Azienda Ospedaliero-Universitaria di Bologna, Bologna, Italy; 6 Department of Diagnostic Imaging, Area of Cardiovascular and Interventional Imaging, Abruzzo Health Unit 1, L’Aquila, Italy; 7 Unit of Diagnostic Imaging, Department of Medicine and Surgery, Santa Maria della Misericordia Hospital, University of Perugia, Perugia, Italy; 8 Radiology Unit, Macerata Hospital, A.S.T, Macerata, Italy; 9 Department of Medical and Surgical Specialties, Radiological Sciences and Public Health, University of Brescia, ASST Spedali Civili of Brescia, Piazzale Spedali Civili, 1, Brescia, I - 25123 Italy; 10 Department of Radiology, Azienda Ospedaliera “G. Brotzu”, Cagliari, Italy; 11 Department of Radiology, “University Hospitals Bristol and Weston Foundation Trust”, Bristol, UK; 12 Diagnostic Radiology Unit Department, CAST - Policlinico - San Marco Hospital University, Catania, Italy; 13 Department of Radiology, “M. G. Vannini” Hospital, Rome, Italy; 14 Unit of Diagnostic and Interventional Imaging, Department of Diagnostic Imaging, Radiation Therapy and Hematology, IRCCS Policlinico “A. Gemelli”, Roma, Italy; 15 Unit of Radiology, Università Cattolica del Sacro Cuore, Roma, Italy; 16 Department of Biomedical Sciences, Humanitas University, Pieve Emanuele, Milan, Italy; 17 IRCCS Humanitas Research Hospital, Rozzano, Milan, Italy

**Keywords:** Surveys and questionnaires, Aortic valve stenosis, Computed tomography angiography, Cardiac imaging techniques

## Abstract

**Purpose:**

The purpose of this survey was to evaluate the current state-of-art of pre-TAVI imaging in a large radiological professional community.

**Methods:**

Between December 2022 and January 2023 all members of the Italian Society of Medical and Interventional Radiology (SIRM) were invited by the CT PRotocol Optimization group (CT-PRO group) to complete an online 24-item questionnaire about pre-TAVI imaging.

**Results:**

557 SIRM members participated in the survey. The greatest part of respondents were consultant radiologists employed in public hospitals and 84% claimed to routinely perform pre-TAVI imaging at their institutions. The most widespread acquisition protocol consisted of an ECG-gated CT angiography (CTA) scan of the aortic root and heart followed by a non-ECG-synchronized CTA of the thorax, abdomen, and pelvis. Contrast agent administration was generally tailored on the patient’s body weight with a preference for using high concentration contrast media. The reports were commonly written by radiologists with expertise in cardiovascular imaging, and included all the measurements suggested by current guidelines for adequate pre-procedural planning. About 60% of the subjects affirmed that the Heart Team is present at their institutions, however only 7% of the respondents regularly attended the multidisciplinary meetings.

**Conclusions:**

This survey defines the current pre-TAVI imaging practice in a large radiological professional community. Interestingly, despite the majority of radiologists follow the current guidelines regarding acquisition and reporting of pre-TAVI imaging studies, there is still a noteworthy absence from multidisciplinary meetings and from the Heart Team.

**Supplementary Information:**

The online version contains supplementary material available at 10.1007/s10554-024-03052-8.

## Introduction

Valvular aortic stenosis is a condition that affects 3–5% of the population over 75 years of age, with a prevalence that is constantly increasing [1–4]. Patients with severe stenosis and symptomatic generally require to undergo elective surgical aortic valve replacement; however, pre-existing comorbidities as well as high preoperative risk may hamper the surgical treatment.

Transcatheter aortic valve implantation (TAVI) has been originally introduced as an alternative to treat selected high-risk patients with severe aortic stenosis, due to its minimally invasive approach [5]. Good outcomes from several trials have shown that TAVI is not inferior to surgical aortic valve replacement, even in younger and lower risk categories of patients. This may lead to a wider adoption of this procedure over the years [6]. A successful procedure requires optimal pre-operative planning, which occurs by evaluation of aortic annulus size, aortic valve structure and calcification, and eligible femoral or epiaortic access sites. In this setting, CT angiography allows to obtain accurate measurements for pre-operative planning, also thanks to increased spatial and temporal resolution of modern CT platforms [7]. However, poor image quality, wrong acquisition protocols and deviations from guidelines may lead to incorrect measurements and potentially fatal periprocedural complications [8, 9]. Recently, the European Society of Cardiovascular Radiology (ESCR) and the Society of Cardiovascular Computed Tomography (SCCT) have published expert consensus documents suggesting the acquisition protocol for pre-TAVI CT angiography, and standardized measurements or nomenclature that should be reported [10].

In this survey we aimed to investigate the current practice for pre-TAVI imaging in a large radiological professional community, and to identify eventual deviations from the ESCR and SCCT recommendations.

## Methods

An online questionnaire was created using the SurveyMonkey platform (www.surveymonkey.com). The survey was conducted from December 22nd 2022 to January 16th 2023 by the CT-Protocol Optimization group (CT-PRO group) and was submitted to about 11.157 radiologists and residents through the mailing list provided by the Italian Society of Medical and Interventional Radiology (SIRM), which endorsed the initiative. The aim was to investigate the state-of-the-art of pre-TAVI imaging amongst a large radiological professional community. The survey consisted of 24 multiple choice questions concerning radiologists’ general characteristics (age, years of experience, job title, type of institution, etc.), the total number of pre-TAVI imaging examinations performed, the scanner and the acquisition protocol characteristics, and the reporting modality. Survey results were summarized with descriptive statistics. The complete list of questions and possible answers of the survey can be found in Appendix.

## Results

### Survey participants characteristics and pre-TAVI imaging activity in Italian centers

**Fig. 1:**
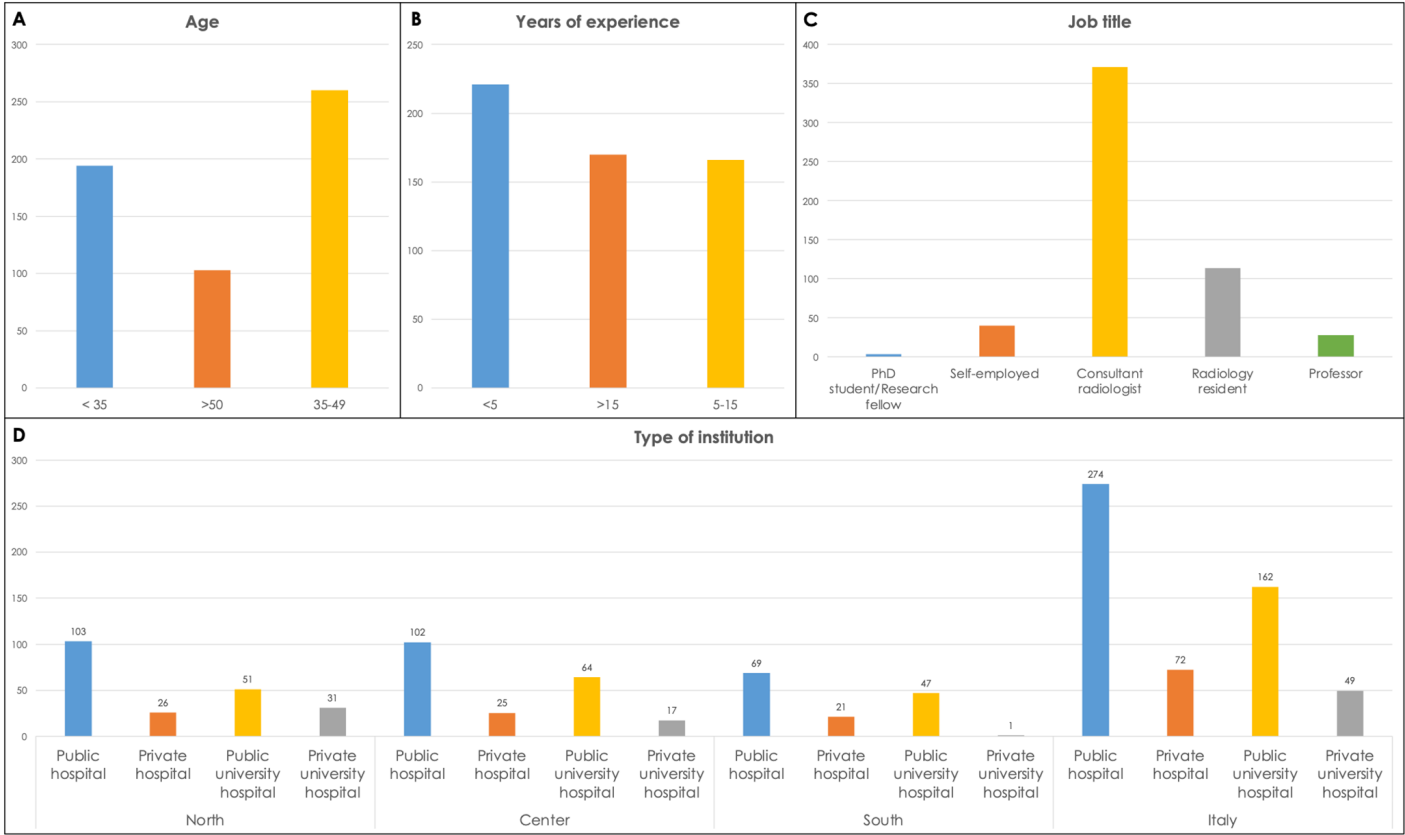
Survey participants characteristics

A total of 557/11.157 (5%) SIRM active members in the year 2022 participated in the survey, but only 333/557 (60%) responded to all the items. The age distribution of respondents was predominantly between 35 and 49 (260/557–47%), with less than 5 years of working experience (221/557–40%). Most of the respondents worked as a consultant (371/557–67%) or resident (114/557–20%) in public institutions (274/557–49%) and public university hospital (162–29%), with a quite homogeneous distribution between North (211/557–38%), Centre (208/557–37%) and South of Italy (138/557–25%). Figure 1 summarizes demographic characteristics of the respondents.

**Fig. 2:**
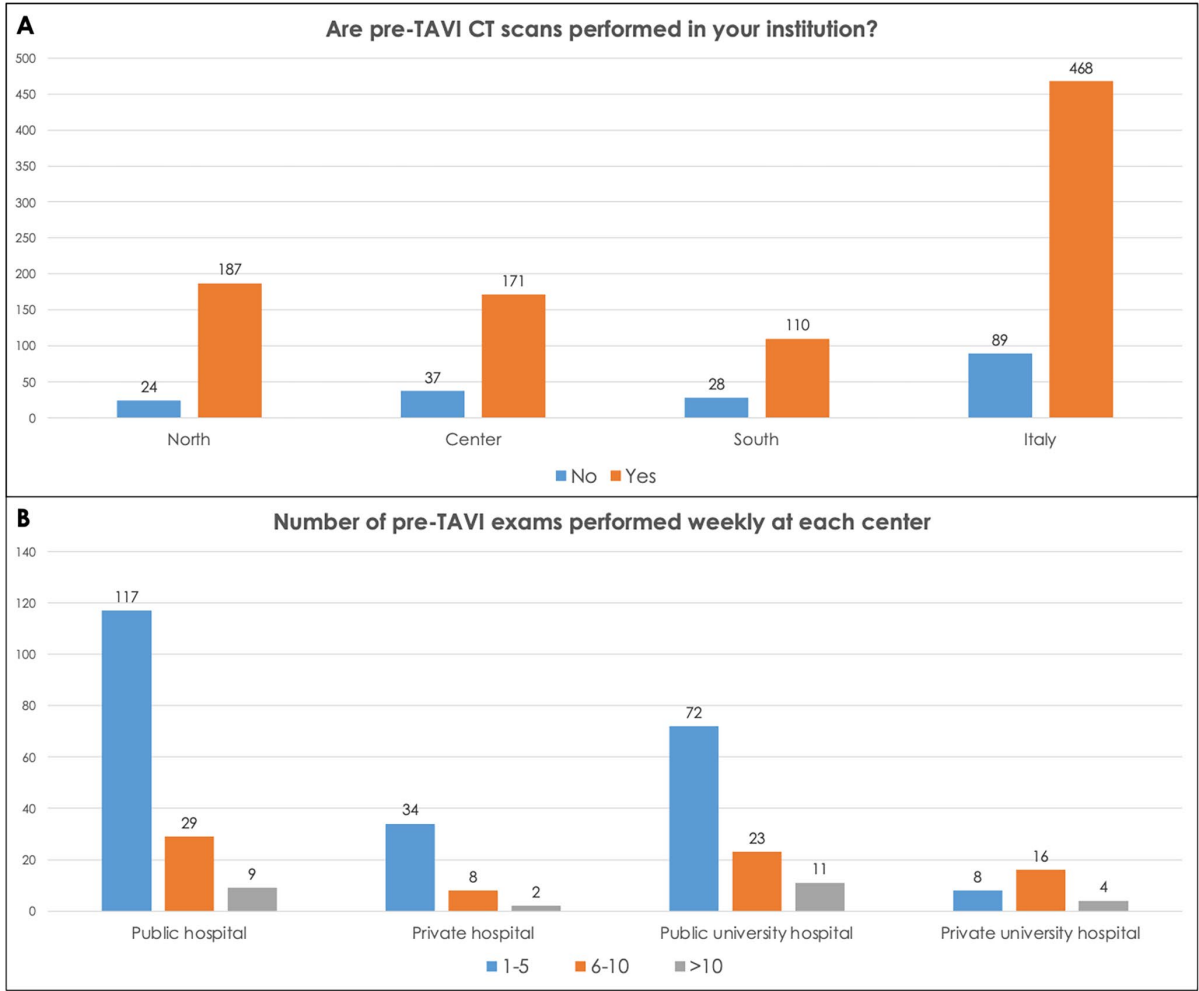
Pre-TAVI imaging activity in Italian centers. **(A)** Institutions where pre-TAVI CT scans are performed, divided by location. **(B)** Number of pre-TAVI studies weekly performed, divided by the type of institution

**Fig. 3:**
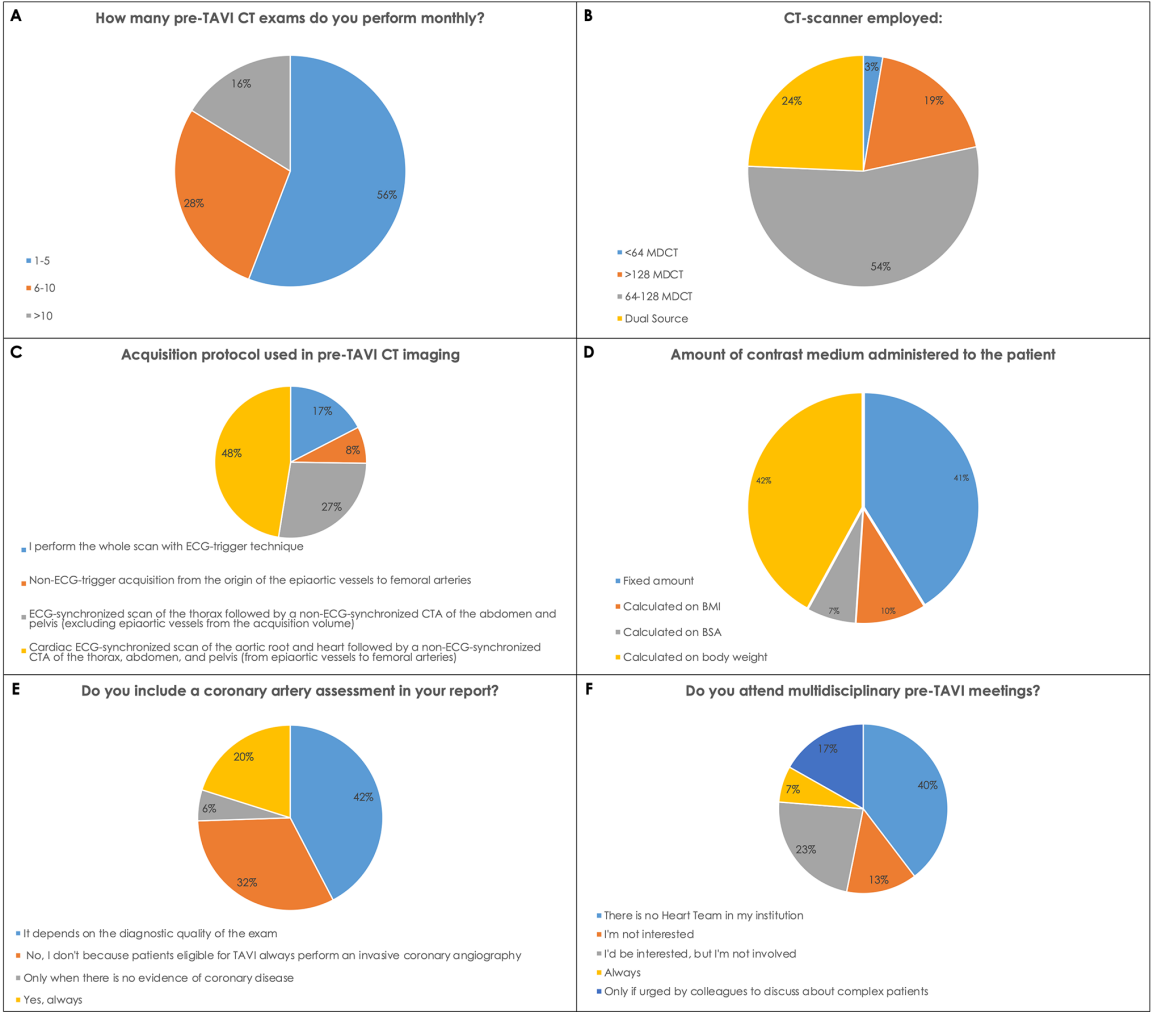
Pre-TAVI imaging acquisition and reporting information

84% (468/557) of respondents affirmed that pre-TAVI CT scans are performed at their institution and most of these centers schedule these investigations into dedicated sessions (221/557–66%). The number of examinations weekly performed in the majority of centers ranges between 1 and 5 (231 − 69%), with a range of 1–5 pre-TAVI imaging reports monthly made by most of the subjects (186–56%) (Fig. 3A). 31% (175/557) of respondents claimed to be the referent radiologist for pre-TAVI imaging at their institution, 28% (158/557) report these studies despite not being his area of expertise, 154/557 (28%) respondents declared they never reported pre-TAVI CT examinations and 13% (70/557) stated that they no longer do it. The number of pre-TAVI imaging examinations performed in private and public hospitals, was overall homogenous. On the other hand, the number of pre-TAVI scans was slightly superior in Northern Italy, followed by Center and South of Italy (Fig. 2). Similarly, the respondents who self-defined experts in cardiovascular imaging were less in South of Italy (20% − 27/138) – than Center and Northern Italy, (30% − 63/208, and 40% − 85/211 respectively).

### Imaging acquisition protocol

More than half of the survey participants use 64–128 slices CT scanners (180–54%) and only 24% (81/333) have dual-energy or spectral CT scanners (Fig. 3B).

Almost all subjects do ECG-synchronized scans. In particular, 158/333 (47%) acquire a cardiac ECG-gated scan of the aortic root and heart followed by a non-ECG-gated CTA of the chest, abdomen, and pelvis, including epiaortic vessels and femoral arteries. 91/333 (27%) acquire an ECG-gated scan of the chest followed by a non-ECG-gated CTA of the abdomen and pelvis but excluding epiaortic vessels from the acquisition volume. 58/333 (17%) of respondents regularly use ECG-gating for the entire acquisition, while only 8% scan patients without any ECG-gating (Fig. 3C).

About contrast agent administration, more than half of the participants use tailored protocols (205/333–62%). Predominantly high concentration contrast agents (> 350 mgI/ml) are used to achieve better visualization of the vascular structures (203–61%). The tailored dose is evaluated on the basis of patients’ body weight (mL/kg) by 42% (140/333) of respondents, on BMI by 10% (33/333), and on BSA by 7% (23/333). The 41% (137/333) gives a fixed dose of contrast agent (Fig. 3D).

In patients with chronic kidney disease (CKD) and reduced estimated glomerular filtration rate (eGFR), more than half of respondents (194/333 − 58%) continue with the administration of the contrast agent at standard doses after premedication and/or nephrological counseling. 19% (65/333) of respondents decrease contrast medium concentration, meanwhile 18% (60/333) reduce the volume, and 15% (51/333) do not proceed with the CTA scan.

The vast majority of respondents (480/557 − 86%) do not perform cardiac magnetic resonance (CMR) as pre-TAVI imaging modality in patients affected by aortic valve stenosis.

### pre-TAVI CT reporting

Pre-TAVI reporting was made by a radiologist with expertise in cardiovascular imaging in 72% of cases (239/333), who provide all the necessary measurements for the procedural planning in 88% of cases (292/333).

Coronary artery assessment is routinely performed in 20% of cases (67/333), while the most of respondents (141/333–42%) report on the coronary tree status only if the image quality is adequate (Fig. 3E).

### Heart team

When asked about participation in multidisciplinary meetings 201/333 (60%) of respondents affirmed that the Heart Team is present at their medical center and of these only 7% (23/333) always takes part in the discussions, while 17% (56/333) only when urged by the other heart team components.

Twenty-three (77/333) of radiologists said not to be involved or invited despite their will to participate, while 14% (45/333) replied not to be interested (Fig. 3F). Interestingly, 40% of the interviewed radiologists answered that the Heart Team is not present at their centers or that they are not aware about its active presence. However, 76% (425/557) of participants affirmed that they would be interested in attending training seminars on pre-TAVI imaging.

## Discussion

Over the last decades, technological innovations led to a net growth of the number of TAVI procedures [11]. Concomitantly, CT angiography has become pivotal for pre-TAVI planning, allowing to obtain all the necessary measurements to select a custom implantable device, as well as assessing valve anatomy and available access routes [12].

In this setting, pre-TAVI imaging may represent the paradigm where the importance of radiological contribution remains controversial, since clinical decision making and procedural planning completely rely on the Heart Team.

### Survey participants characteristics and pre-TAVI imaging activity in Italian centers

The current situation in a large Italian radiological professional community, shows that most of the radiologists involved in pre-TAVI imaging studies are specialists in cardiovascular imaging with less than 5-years of experience, who primarily work as consultants in national health system hospitals. This data could be related to the exponential growth recently observed in cardiovascular CT and MRI examinations, as well as related to the increase of TAVI procedures demanding such examinations. However, only 18% of respondents to this survey were older than 50-years-old, and this may contribute to a bias of this result. Results also showed that most of the Italian institutes routinely perform imaging for pre-procedural TAVI planning with a fairly homogeneous distribution in the whole Country, despite the number is slightly superior in Northern Italy, compared to Center and South of Italy.

### Imaging acquisition protocol

According to the SCCT guidelines, two different approaches for the appropriate acquisition of pre-TAVI imaging should be performed by CT angiography. The first approach requires a cardiac ECG-gated dataset of the aortic root and heart and a non-ECG-gated scan including thorax, abdomen, and pelvis. Alternatively, the second option is an ECG-gated scan of the thorax followed by a non-ECG-gated acquisition of the abdomen and pelvis [10]. CT scanner technology with a minimum of 64-detectors is considered to be necessary for optimal image acquisition [2]. The minimal technology equipment required for the execution of pre-TAVI CT angiography is not achieved by 3% of respondents. However, our results have shown that only 74% of respondents follow the CT acquisition approaches suggested by current guidelines. On the other hand, 8% of respondents do not use ECG-gating in their pre-TAVI CT scans, which may significantly hamper the final image quality. Of these, none has answered to perform more than five scans per month.

For what concerns the contrast agent administration, current consensus documents recommend to use high flows and a dosage that generally ranges between 50 and 100 ml, to be preferably administered via antecubital intravenous access [10].

The survey showed that the majority of respondents tend to favor the use of high concentration contrast media, and that they adjust contrast volumes on the basis of patients’ weight to obtain optimal arterial visualization [13]. This choice can be explained by the need to obtain better average results in a wide variety of patients, including obese or overweight ones and those with low stroke volume.

Patients suffering from aortic stenosis are often elderly and frail, affected by multiple comorbidities. The presence of concomitant CKD and eGFR values less than 30 ml/min/1.73m^2^ may lead to post-contrast acute kidney injury (PC-AKI) [14, 15]. In this category of patients, the majority of our survey respondents claimed to proceed with the administration of standard doses of contrast medium after a proper premedication and nephrological consultation.

In this setting, current guidelines suggest diminishing the dosage and adopting different stratagems (e.g. lower flow rates, low tube potential, etc.) that help maintain an adequate attenuation of the vessels and minimizing the risk for PC-AKI. Several studies have shown that lower volumes of contrast agent, associated with kVp reduction, still allow to achieve sufficient pre-TAVI imaging evaluation. In particular, Kok at al. demonstrated that the reduction between 34 and 67% of contrast medium amount still gave a satisfactory visualization of the vascular structures [16]. In addition, in centers where this technology is available, dual-energy or spectral CT scanners may benefit from virtual monoenergetic reconstructions that improve image quality and vascular attenuation in cases of poor contrast condition [17–19].

The use of CMR for pre-TAVI imaging assessment is not common in patients with aortic stenosis. However, CMR is a valuable tool for determining the severity of valve injury, as well as to obtain valvular or cardiac functional information, especially in cases where trans-thoracic or trans-esophageal echocardiography provide inconclusive results. Our survey results showed that 10% of radiologists perform CMR, mostly to assess the presence of myocardial fibrosis or amyloidosis. CMR has also been described to be able to assess all pre-TAVI measurements [20]. However, CT remains the method of choice for pre-TAVI planning considering its higher spatial resolution and ease of execution, which makes this technique much better tolerated by frail patients.

### pre-TAVI CT reporting

The information enclosed in the radiological report is essential for the planning and the success of TAVI procedure. The complete description of the valvular aortic measurements is mandatory, and these should be recorded as key-images or screenshots; in addition, valve characteristics and patency and characteristics of arterial access sites, such as the presence of stenoses or the possible presence of severe arterial kinkings, should always be discussed in the report.

The evaluation of the coronary arteries in the context of a pre-TAVI CT angiography is still controversial, even because invasive coronary angiography is usually performed prior to TAVI procedure. However, the description of any visible coronary artery stenosis or plaque burden, as well as the anatomical variants, should always be reported.

Despite several studies having demonstrated the feasibility of a concomitant coronary assessment, many of the survey respondents claimed to perform the coronary assessment exclusively in relation to the quality of the acquired datasets. However, image quality in pre-TAVI CT angiography may often be reduced due to the limitations in drugs administration for adequate exam preparation (e.g. beta-blockers, glyceryl-trinitrate) in this category of patients [12, 21–25].

### Heart team

The presence of a Heart Team in centers performing TAVI procedures ensures a constant interaction between interventional cardiologists, cardiac surgeons and cardiovascular radiologists, who are responsible to select the optimal therapeutic option for each patient based on shared decision-making process, especially for those “gray areas” where the choice between surgical approach and TAVI is not clearly defined. Usually, this decision should be carefully assessed on the basis of age, clinical conditions of patients, life expectancy, and pre-operative risk [26, 27]. Interestingly, the results coming from our survey show that Heart Team is absent in most of the Italian responding institutes, or that the involvement of radiologists in multidisciplinary discussions is deficient.

Finally, we also found that the interest for cardiovascular imaging has been growing considerably in recent years. 76% of the respondents declared they are interested in attending dedicated seminars. Indeed, the availability of training courses, especially among young radiologists and residents, would help increase the number of imaging experts able to perform pre-TAVI investigations.

Our results have several limitations. Firstly, the data produced in a survey generally lack details or depth on the topic being investigated and this might be particularly true when response rate is relatively low, such as in our case. In fact, the radiological professional community involved in this survey constitutes a relatively small portion of the Italian radiologists (about 5% of SIRM members). This is probably due to the prevalence of members who have more interest in this specific topic, and might not be representative of the entire Italian radiologists or might not reveal the general approach to pre-TAVI imaging used by the majority of them. Moreover, the survey only obtained responses from SIRM members, meaning that an unknown number of Italian radiologists have not been reached. Other analogous studies found similar limitations that could be responsible for bias [28–31]. Furthermore, there was a low prevalence of PhD students, research fellows (1%) and professors (5%) among respondents, as well as from private institutes’ employees (22%), that could have led to significant discrepancies in the responses, focusing the attention more on the protocols and the approach adopted over public centers. Additionally, our data represent real-world observations on a representative sample, without statistical comparison between the different groups, nor between the responses assessed on the basis of parameters such as age or years of experience of the respondents, or on the volume of examinations performed at a given center. Finally, in order to keep the length of the questionnaire at minimum, some important aspects concerning the CT acquisition protocol (e.g. retrospective vs. prospective ECG-gating) or contrast agent administration may have not been sufficiently explored, leaving room for further investigation.

In conclusion, this survey shows that pre-TAVI imaging has been increasingly performed in Italian hospitals, with a comparable trend to that of TAVI procedures. However, radiologists who are currently dedicated to these investigations or who have expertise in cardiovascular imaging may not be enough. It follows that in order to deal with the increasing number of pre-TAVI CT scans in the coming years, it might be expected to train a greater number of professionals.

### Electronic supplementary material

Below is the link to the electronic supplementary material.

Supplementary Material 1

Supplementary Material 2

## Data Availability

No datasets were generated or analysed during the current study.
