# Supplementary material for: Pre-TAVI imaging: an Italian survey by the CT PRotocol optimization (CT-PRO) group
Source: Int J Cardiovasc Imaging. 2024 Jan 24;40(4):831–9. doi: 10.1007/s10554-024-03052-8 (PMC11052815; doi:10.1007/s10554-024-03052-8)
Supplement: Supplementary file 1 — Supplementary Material 1 [file 10554_2024_3052_MOESM1_ESM.pdf]

Pre-TAVI Imaging: an Italian survey by the CT PRotocol Optimization (CT-PRO) group

The International Journal of Cardiovascular Imaging

Tommaso D'Angelo, Ludovica R. M. Lanzafame, Carlo Liguori, Cesare Mantini, Vincenzo Russo, Pierpaolo Palumbo, Giovanni B. Scalera, Andrea Iozzelli, Andrea Borghesi, Gildo Matta, Fabio Greco, Valeria Garretto, Massimiliano Danti, Roberto Iezzi, Marco Francone

Corresponding Author:
Dr. Tommaso D'Angelo, MD, PhD
Unit of Diagnostic and Interventional Imaging, Department BIOMORF, University of Messina, Messina, Italy
(tommasodang@gmail.com)

APPENDIX

1. Age (years)

A.	< 35	194/557	35%
B.	>50	103/557	18%
C.	35-49	260/557	47%

2. Years of experience

A.	<5	221/557	40%
B.	>15	170/557	31%
C.	5-15	166/557	30%

3. Job title

A.	PhD student/Research fellow	5/557	1%
B.	Self-employed	40/557	7%
C.	Consultant radiologist	371/557	67%
D.	Radiology resident	114/557	20%
E.	Professor	28/557	5%

4. Type of institution

A.	Public hospital	274/557	49%
B.	Private hospital	72/557	13%
C.	Public university hospital	162/557	29%
D.	Private university hospital	49/557	9%

5. Location (in Italy)

A.	Center	208/557	37%
B.	North	211/557	38%
C.	South (and islands)	138/557	25%

6. Are pre-TAVI CT scans performed at your center?

A.	No	89/557	16%
B.	Yes	468/557	84%

7. Do you perform/have you performed pre-TAVI CT scans?

A.	Not currently, but I used to	70/557	13%
B.	No, I’ve never done it	154/557	28%
C.	Yes, but it’s not my main activity/interest	158/557	28%
D.	Yes, I am one of the referents of my center for these studies	175/557	31%

8. Why don’t you perform pre-TAVI CT studies?

A.	At my institution there are no requests for pre-TAVI CT examinations	75/224	33%
B.	I'm not interested	33/224	15%
C.	I’d be interested but I don’t have the expertise	48/224	21%
D.	They are reported by other specialists	68/224	30%

9. How many pre-TAVI CT exams are performed weekly at your center?

A.	>10	26/333	8%
B.	1-5	231/333	69%
C.	6-10	76/333	23%

10. How many pre-TAVI CT exams do you perform monthly?

A.	>10	54/333	16%
B.	1-5	186/333	56%
C.	6-10	93/333	28%

11. CT-scanner employed:

A.	< 64 MDCT	9/333	3%
B.	>128 MDCT	63/333	19%
C.	64-128 MDCT	180/333	54%
D.	DECT	81/333	24%

12. Are pre-TAVI CTs scheduled on dedicated sessions?

A.	No	112/333	34%
B.	Yes	221/333	66%

13. What acquisition protocol do you generally use in pre-TAVI CT imaging?

A.	I scan with ECG-synchronization, from the origin of the epiaortic vessels to femoral arteries	58/333	17%
B.	I scan without ECG-synchronization, from the origin of the epiaortic vessels to femoral arteries	26/333	8%
C.	ECG-synchronized scan of the thorax followed by a non-ECG-synchronized CTA of the abdomen and pelvis (excluding epiaortic vessels from the acquisition volume)	91/333	27%
D.	ECG-synchronized scan of the aortic root and heart followed by a non-ECG-synchronized CTA of the thorax, abdomen, and pelvis (from epiaortic vessels to femoral arteries)	158/333	47%

14. Do you use tailored protocols for contrast agent administration for pre-TAVI CTs?

A.	No	128/333	38%
B.	Yes	205/333	62%

15. For pre-TAVI CT tests, which of the following statements corresponds to your clinical practice for contrast agent administration?

A.	I adjust the protocol to the preset IDR value	50/333	15%
B.	The choice of contrast agent does not affect the quality of the study	35/333	11%
C.	I don't administer contrast agent in patients at risk	2/333	1%
D.	I mainly use high concentration contrast medium (>350 mgI/mL) to obtain higher vascular enhancement	203/333	61%
E.	I mainly use low concentration contrast medium (<350 mgI/mL) because of the fragility of patients who undergo pre-TAVI CT studies	43/333	13%

16. For pre-TAVI CT tests, the amount of contrast medium administered to the patient ____ :

A.	It is usually a fixed amount	137/333	41%
B.	It is calculated on BMI	33/333	10%
C.	It is calculated on BSA	23/333	7%
D.	It is calculated on body weight	140/333	42%

17. Which of the following statements describes your clinical practice for contrast agent administration in patient at risk for PC-AKI (eGFR less than 30 ml/min/1.73m^2)?

A.	I do the scan with the administration of standard dose of contrast agent after premedication and/or nephrological counseling	194/333	58%
B.	I do the scan without contrast medium administration	9/333	3%
C.	I do not scan the patient	51/333	15%
D.	I do the scan, but I reduce the contrast medium volume	60/333	18%
E.	I do the scan, but I decrease the contrast medium concentration	19/333	6%

18. Which of the following statements describes your report for pre-TAVI CT studies?

A.	I provide a detailed description only of access sites. Measurements of the aortic valve are done by cardiologists	12/333	4%
B.	I provide all the measurements necessary for procedural planning	292//333	88%
C.	I report exclusively thoracic and abdominal findings without vascular measurements	6//333	2%
D.	B+C	16/333	5%
E.	I report thoracic and abdominal vessels findings without the necessary measurements	7/333	2%

19. Do you include a coronary artery assessment in your report?

A.	It depends on the diagnostic quality of the exam	141/333	42%
B.	No, I don't because patients eligible for TAVI always perform an invasive coronary angiography	107/333	32%
C.	Only when there is no evidence of coronary disease	18/333	5%
D.	Yes, always	67/333	20%

20. Do you attend multidisciplinary pre-TAVI meetings?

A.	There is no Heart Team in my institution	132/333	40%
B.	I'm not interested	45/333	14%
C.	I'd be interested, but I'm not involved	77/333	23%
D.	Always	23/333	7%
E.	Only if urged by colleagues to discuss about complex patients	56/333	17%

21. Who reports pre-TAVI CT studies in your institution?

A.	Every radiologist	73/333	22%
B.	Only radiologists with expertise in cardiovascular imaging	239/333	72%
C.	There is no dedicated figure	1/333	0%
D.	Radiologist and cardiologist (together)	9/333	3%
E.	Radiologist or cardiologist	1/333	0%
F.	Other	10/333	3%

- Sometimes they are erroneously scheduled in non-dedicated sessions, but generally they reported by dedicated radiologists
- I report pre-TAVI CT studies
- My colleague and I, we are designated as reference persons for this kind of examination, because of our interest in cardiovascular imaging
- I work in a highly specialized center dedicated to cardiac imaging
- The pre-TAVI CT study is reported by interventional radiologists and the pre-TAVI CT study + CCTA by general radiologists
- Neuroradiologists
- We are all specialists in cardiovascular imaging
- Only radiologists with expertise in cardiovascular imaging
- Usually cardiovascular radiologists, otherwise the radiologist on duty
- Only me

22. Do you perform CMR in TAVI candidates?

A.	Never	480/557	86%
B.	Yes, to achieve a better functional characterization of aortic stenosis	19/557	3%
C.	Yes, to assess myocardial fibrosis/amyloidosis	35/557	6%
D.	Only in case of relative contraindications to CT	23/557	4%

23. If you have positively answered to the previous question, what percentage of patients undergo CMR?

A.	5-10%	13/77	17%
B.	<5%	22/77	29%
C.	<2%	42/77	55%

24. Would you be interested in pre-TAVI imaging seminars?

A.	No	132/557	24%
B.	Yes	425/557	76%
