# Supplementary material for: Pre-TAVI imaging: an Italian survey by the CT PRotocol optimization (CT-PRO) group
Source: Int J Cardiovasc Imaging. 2024 Jan 24;40(4):831–9. doi: 10.1007/s10554-024-03052-8 (PMC11052815; doi:10.1007/s10554-024-03052-8)
Supplement: Supplementary file 2 — Supplementary Material 2 [file 10554_2024_3052_MOESM2_ESM.docx]

**Pre-TAVI Imaging: an Italian survey by the CT PRotocol Optimization (CT-PRO) group**

*The International Journal of Cardiovascular Imaging*

Tommaso D’Angelo, Ludovica R. M. Lanzafame, Carlo Liguori, Cesare Mantini, Vincenzo Russo, Pierpaolo Palumbo, Giovanni B. Scalera, Andrea Iozzelli, Andrea Borghesi, Gildo Matta, Fabio Greco, Valeria Garretto, Massimiliano Danti, Roberto Iezzi, Marco Francone

**Corresponding Author:**

Dr. Tommaso D’Angelo, MD, PhD

Unit of Diagnostic and Interventional Imaging, Department BIOMORF, University of Messina, Messina, Italy

(tommasodang@gmail.com)

**APPENDIX**

| **1. Age (years)**   1. < 35 2. >50 3. 35-49 | 194/557 103/557  260/557 | 35%  18%  47% |
| --- | --- | --- |
|  |  |  |
| **2. Years of experience**   1. <5 2. >15 3. 5-15 | 221/557 170/557 166/557 | 40%  31%  30% |
|  |  |  |
| **3. Job title**   1. PhD student/Research fellow 2. Self-employed 3. Consultant radiologist 4. Radiology resident 5. Professor | 5/557 40/557 371/557 114/557 28/557 | 1%  7%  67%  20%  5% |
|  |  |  |
| **4. Type of institution**   1. Public hospital 2. Private hospital 3. Public university hospital 4. Private university hospital | 274/557 72/557 162/557 49/557 | 49%  13%  29%  9% |
|  |  |  |
| **5. Location (in Italy)**   1. Center 2. North 3. South (and islands) | 208/557 211/557 138/557 | 37%  38%  25% |
|  |  |  |
| **6. Are pre-TAVI CT scans performed at your center**?   1. No 2. Yes | 89/557  468/557 | 16%  84% |
|  |  |  |
| **7. Do you perform/have you performed pre-TAVI CT scans?**   1. Not currently, but I used to 2. No, I’ve never done it 3. Yes, but it’s not my main activity/interest 4. Yes, I am one of the referents of my center for these studies | 70/557 154/557  158/557 175/557 | 13%  28%  28%  31% |
|  |  |  |
| **8. Why don’t you perform pre-TAVI CT studies?**   1. At my institution there are no requests for pre-TAVI CT examinations 2. I'm not interested 3. I’d be interested but I don’t have the expertise 4. They are reported by other specialists | 75/224 33/224  48/224 68/224 | 33%  15%  21%  30% |
|  |  |  |
| **9. How many pre-TAVI CT exams are performed weekly at your center?**   1. >10 2. 1-5 3. 6-10 | 26/333  231/333  76/333 | 8%  69%  23% |
|  |  |  |
| **10. How many pre-TAVI CT exams do you perform monthly?**   1. >10 2. 1-5 3. 6-10 | 54/333 186/333 93/333 | 16%  56%  28% |
|  |  |  |
| **11. CT-scanner employed:**   1. < 64 MDCT 2. >128 MDCT 3. 64-128 MDCT 4. DECT | 9/333  63/333 180/333 81/333 | 3%  19%  54%  24% |
|  |  |  |
|  |  |  |
| **12. Are pre-TAVI CTs scheduled on dedicated sessions?**   1. No 2. Yes | 112/333 221/333 | 34%  66% |
|  |  |  |
| **13. What acquisition protocol do you generally use in pre-TAVI CT imaging?**   1. I scan with ECG-synchronization, from the origin of the epiaortic vessels to femoral arteries 2. I scan without ECG-synchronization, from the origin of the epiaortic vessels to femoral arteries 3. ECG-synchronized scan of the thorax followed by a non-ECG-synchronized CTA of the abdomen and pelvis (excluding epiaortic vessels from the acquisition volume) 4. ECG-synchronized scan of the aortic root and heart followed by a non-ECG-synchronized CTA of the thorax, abdomen, and pelvis (from epiaortic vessels to femoral arteries) | 58/333 26/333  91/333  158/333 | 17%  8%  27%  47% |
|  |  |  |
| **14. Do you use tailored protocols for contrast agent administration for pre-TAVI CTs?**   1. No 2. Yes | 128/333 205/333 | 38%  62% |
|  |  |  |
| **15. For pre-TAVI CT tests, which of the following statements corresponds to your clinical practice for contrast agent administration?**   1. I adjust the protocol to the preset IDR value 2. The choice of contrast agent does not affect the quality of the study 3. I don’t administer contrast agent in patients at risk 4. I mainly use high concentration contrast medium (>350 mgI/mL) to obtain higher vascular enhancement 5. I mainly use low concentration contrast medium (<350 mgI/mL) because of the fragility of patients who undergo pre-TAVI CT studies | 50/333  35/333 2/333  203/333  43/333 | 15% 11% 1%  61%  13% |
|  |  |  |
| **16. For pre-TAVI CT tests, the amount of contrast medium administered to the patient ____ :**   1. It is usually a fixed amount 2. It is calculated on BMI 3. It is calculated on BSA 4. It is calculated on body weight | 137/333 33/333 23/333 140/333 | 41%  10%  7%  42% |
|  |  |  |
| **17. Which of the following statements describes your clinical practice for contrast agent administration in patient at risk for PC-AKI (eGFR less than 30 ml/min/1.73m^2^)?**   1. I do the scan with the administration of standard dose of contrast agent after premedication and/or nephrological counseling 2. I do the scan without contrast medium administration 3. I do not scan the patient 4. I do the scan, but I reduce the contrast medium volume 5. I do the scan, but I decrease the contrast medium concentration | 194/333 9/333 51/333 60/333 19/333 | 58%  3%  15%  18%  6% |
|  |  |  |
|  |  |  |
|  |  |  |
|  |  |  |
| **18. Which of the following statements describes your report for pre-TAVI CT studies?**   1. I provide a detailed description only of access sites. Measurements of the aortic valve are done by cardiologists 2. I provide all the measurements necessary for procedural planning 3. I report exclusively thoracic and abdominal findings without vascular measurements 4. B+C 5. I report thoracic and abdominal vessels findings without the necessary measurements | 12/333 292//333 6//333 16/333 7/333 | .  4%  88%  2%  5%  2% |
|  |  |  |
| **19. Do you include a coronary artery assessment in your report?**   1. It depends on the diagnostic quality of the exam 2. No, I don’t because patients eligible for TAVI always perform an invasive coronary angiography 3. Only when there is no evidence of coronary disease 4. Yes, always | 141/333 107/333 18/333 67/333 | 42%  32%  5%  20% |
|  |  |  |
| **20. Do you attend multidisciplinary pre-TAVI meetings?**   1. There is no Heart Team in my institution 2. I'm not interested 3. I’d be interested, but I’m not involved 4. Always 5. Only if urged by colleagues to discuss about complex patients | 132/333 45/333 77/333 23/333 56/333 | 40%  14%  23%  7%  17% |
|  |  |  |
| **21. Who reports pre-TAVI CT studies in your institution?**   1. Every radiologist 2. Only radiologists with expertise in cardiovascular imaging 3. There is no dedicated figure 4. Radiologist and cardiologist (together) 5. Radiologist or cardiologist 6. Other  - Sometimes they are erroneously scheduled in non-dedicated sessions, but generally they reported by dedicated radiologists - I report pre-TAVI CT studies - My colleague and I, we are designated as reference persons for this kind of examination, because of our interest in cardiovascular imaging - I work in a highly specialized center dedicated to cardiac imaging - The pre-TAVI CT study is reported by interventional radiologists and the pre-TAVI CT study + CCTA by general radiologists - Neuroradiologists - We are all specialists in cardiovascular imaging - Only radiologists with expertise in cardiovascular imaging - Usually cardiovascular radiologists, otherwise the radiologist on duty - Only me | 73/333 239/333 1/333 9/333 1/333 10/333 | 22% 72%  0%  3%  0%  3% |
|  |  |  |
| **22. Do you perform CMR in TAVI candidates?**   1. Never 2. Yes, to achieve a better functional characterization of aortic stenosis 3. Yes, to assess myocardial fibrosis/amyloidosis 4. Only in case of relative contraindications to CT | 480/557 19/557 35/557 23/557 | 86%  3%  6% 4% |
|  |  |  |
| **23. If you have positively answered to the previous question, what percentage of patients undergo CMR?**   1. 5-10% 2. <5% 3. <2% | 13/77 22/77 42/77 | 17%  29%  55% |
|  |  |  |
| **24. Would you be interested in pre-TAVI imaging seminars?**   1. No 2. Yes | 132/557 425/557 | 24%  76% |
